# The origins of acoustic communication in vertebrates

**DOI:** 10.1038/s41467-020-14356-3

**Published:** 2020-01-17

**Authors:** Zhuo Chen, John J. Wiens

**Affiliations:** 1 College of Life Sciences, Henan Normal University, 453007 Xinxiang, Henan Province China; 2 Department of Ecology and Evolutionary Biology, University of Arizona, Tucson, AZ 85721-0088 USA

**Keywords:** Evolutionary ecology, Social evolution, Speciation, Animal behaviour

## Abstract

Acoustic communication is crucial to humans and many other tetrapods, including birds, frogs, crocodilians, and mammals. However, large-scale patterns in its evolution are largely unstudied. Here, we address several fundamental questions about the origins of acoustic communication in terrestrial vertebrates (tetrapods), using phylogenetic methods. We show that origins of acoustic communication are significantly associated with nocturnal activity. We find that acoustic communication does not increase diversification rates, a surprising result given the many speciation-focused studies of frog calls and bird songs. We also demonstrate that the presence of acoustic communication is strongly conserved over time. Finally, we find that acoustic communication evolved independently in most major tetrapod groups, often with remarkably ancient origins (~100–200 million years ago). Overall, we show that the role of ecology in shaping signal evolution applies to surprisingly deep timescales, whereas the role of signal evolution in diversification may not.

## Abstract (web summary)

Acoustic communication is widespread, but not universal, across terrestrial vertebrates. Here, the authors show that acoustic communication evolved anciently, but independently, in most tetrapod groups and that these origins were associated with nocturnal activity.

## Introduction

Acoustic communication is widespread and important in many terrestrial vertebrates (tetrapods), including amphibians (e.g., frogs), mammals, and reptiles (e.g., geckos, birds, crocodilians). For example, acoustic signals appear to be widely used in mate choice in frogs and birds and are crucial for sexual selection, species recognition, and speciation in these groups^1^. Many aspects of acoustic communication have now been intensely studied in vertebrates, especially given the importance of acoustic communication in humans and its potential role in the evolution of language^2^. However, research on the evolution of acoustic communication has generally focused on variation in acoustic signals within species and among closely related species^1,3–5^ or within clades^6–9^.

In contrast, many fundamental questions about the origins of acoustic communication in tetrapods have not been explicitly analyzed. First, perhaps most importantly, why did acoustic communication originate? Specifically, what ecological factors explain which lineages have acoustic communication and which do not? For example, it has been hypothesized that acoustic communication is more likely to originate in nocturnal lineages than diurnal lineages^10^. This hypothesis is logical given that acoustic signals can function in darkness, whereas most visual signals cannot^10^. However, to our knowledge, this hypothesis has not been explicitly tested. Second, given the potential role of acoustic communication in driving speciation in different tetrapod groups^1,3–5^, does the presence of acoustic communication in certain clades help explain large-scale patterns of diversification (speciation minus extinction) and species diversity across tetrapods? Third, is the presence of acoustic communication evolutionarily stable once it evolves? Fourth, did acoustic communication evolve independently in different tetrapod groups (e.g., frogs, birds, mammals) or from a common ancestor? If it evolved independently, how old are its origins in each group?

These questions are not only relevant to acoustic communication in vertebrates, but are also relevant to some important and long-standing questions in evolutionary biology and animal behavior. For example, the idea that the environment where species occur shapes their signal evolution has been very influential in the study of sexual selection and animal communication^11,12^. However, this idea is generally applied to differences in a given signal type among closely related species (e.g., color among closely related fish species^13^), typically in different habitats. Here we test whether this idea applies to basic signal types (i.e., acoustic) over timescales of hundreds of millions of years and where the signaling environment is based on diel activity, not habitat. Similarly, the idea that sexually selected traits (such as acoustic signals) drive speciation has been prominent in the evolutionary literature ever since Darwin^13–21^. Yet, support for a strong relationship between specific traits and accelerated diversification rates has been surprisingly mixed^17–20^. Here we test the idea that a sexually selected trait (with particularly well-established links to speciation) can drive diversification at relatively deep phylogenetic scales. Finally, to our knowledge, no previous studies have tested the evolutionary stability of overall signal types (e.g., acoustic). In summary, questions about the evolution of acoustic communication in tetrapods have relevance that goes beyond this particular trait and specific group of organisms.

In this study, we address the evolution of acoustic communication in tetrapods using a phylogenetic approach. We focus on the overall origins and presence of acoustic communication among species, rather than on the form or function of particular acoustic signals. We utilize time-calibrated phylogenies^22^ for 1799 representative tetrapod species (Supplementary Data 1–2), with species sampled in proportion to the richness of the major clades to which they belong. We obtain data from the literature on the presence and absence of acoustic communication within each sampled species (Supplementary Data 3). We define acoustic communication here as transmission of messages between conspecifics via sound waves through air or water (but excluding signals transmitted only to heterospecifics and only through solids; see “Methods”). We also compile data on the diel (day–night) activity patterns of these same species (Supplementary Data 4). We then use these data to test whether the origins of acoustic communication are associated with nocturnal activity, using phylogenetic methods. Specifically, we utilize a maximum-likelihood test of correlated evolution^23^ and phylogenetic logistic regression^24^. We also test whether the presence of acoustic communication in a lineage increases its rates of species diversification, using Hidden-State Speciation–Extinction (HiSSE) models^25^. We also test for phylogenetic conservatism in the presence of acoustic communication among species^26,27^. Finally, we trace the origins of acoustic communication across tetrapod phylogeny, to address whether acoustic communication arose independently in different groups, and when.

Our results show that the evolution of acoustic communication is associated with nocturnal activity. Thus we support the idea that ecology helps shape signal evolution^11,12^ and show that this idea applies to basic types of signals (i.e. acoustic) over timescales of hundreds of millions of years. In contrast, we show that the presence of acoustic communication does not significantly accelerate diversification over deep timescales, despite the intensive study of the role of calls and songs in speciation^1^. We also find that acoustic communication is strongly conserved phylogenetically and, despite multiple origins across tetrapods, has been maintained in some clades for ~100–200 million years. Thus we show that some types of signal traits can be conserved over surprisingly deep timescales.

## Results

### Distribution of acoustic communication

Across tetrapods, most amphibians, mammals, crocodilians, and birds have acoustic communication, whereas most lepidosaurs and turtles do not (Table 1; Supplementary Data 3). Among amphibians, acoustic communication is absent in caecilians but present in a few salamanders and most frogs (in 39 of 41 families sampled). Acoustic communication is absent in snakes and all but two lizard families (i.e., Gekkonidae, Phyllodactylidae). In turtles, acoustic communication occurs in 2 of the 14 families. Acoustic communication was present in all 173 bird families sampled. In mammals, acoustic communication was present in all 23 orders and 120 of the 125 sampled families. Extrapolating from the species in our sample, we estimate that acoustic communication is present in ~69% of tetrapod species (Table 1).

**Table 1:** Summary data among major clades.

Clade	Acoustic present (%)	Sampled species	Described species
Amphibians	89.0	508	7966
Mammals	94.9	235	6399
Lepidosaurs	3.3	490	10,418
Turtles	18.8	16	351
Crocodilians	100	1	24
Birds	100	549	10,711
Tetrapods	69.2	1799	35,869

Percentage of sampled species with acoustic communication in each major tetrapod clade, along with the total number of sampled species, and the total number of described, extant species in each clade. Data for each species are given in Supplementary Data 3

### Relationships between diel activity and acoustic communication

Prior to testing relationships between acoustic communication and diel activity, we tested the best-fitting model of evolution for each character (Supplementary Tables 1 and 2). Results are shown for the model allowing different rates of gains and losses for both traits (Table 2). We found significant relationships between acoustic communication and diel activity using Pagel’s^23^ likelihood test (*P* < 0.05, *n* = 1799, Table 2; Supplementary Table 3). Acoustic communication was dependent on diel activity or both traits were dependent on each other, but the dependence of diel activity on acoustic communication was not supported (Table 2). Comparison of Akaike Information Criterion (AIC) and weighted AIC (AIC_W_) scores^28^ supported the dependence of acoustic communication on diel activity (Supplementary Table 4). Phylogenetic logistic regression^24^ showed a significant relationship between origins of acoustic communication and nocturnal activity (*P* < 0.05, *n* = 1799, Table 3). All results were consistent using different evolutionary models, alternative trees, and two alternative coding methods for diel activity (Tables 2 and 3; Supplementary Table 3).

**Table 2:** Results of likelihood analyses of correlated evolution between acoustic communication and diel activity.

Coding and tree	Dependent variable	Independent model (AIC)	Dependent model (AIC)	Likelihood ratio	*P*
Maximum diurnal					
Ericson	Acoustic	1667.669	**1656.630**	15.0393	0.0005
	Diel	**1667.669**	1671.599	0.0706	0.9653
	Acoustic and diel	1667.669	**1660.581**	15.0887	0.0045
Hackett	Acoustic	1679.120	**1668.694**	14.4259	0.0007
	Diel	**1679.120**	1682.932	0.1880	0.9103
	Acoustic and diel	1679.120	**1672.662**	14.4581	0.0060
Maximum nocturnal					
Ericson	Acoustic	1396.779	**1384.295**	16.4837	0.0003
	Diel	**1396.779**	1400.731	0.0481	0.9762
	Acoustic and diel	1396.779	**1388.039**	16.7399	0.0022
Hackett	Acoustic	1410.804	**1395.390**	19.4144	0.0001
	Diel	**1410.804**	1414.757	0.0474	0.9766
	Acoustic and diel	1410.804	**1399.124**	19.6802	0.0006

Analyses were conducted using two methods for coding day–night activity patterns in arrhythmic and crepuscular species (maximum diurnal and maximum nocturnal) and two different trees (Ericson^29^ vs. Hackett^30^ backbone trees for birds). Three dependent models were tested: (i) acoustic communication depends on diel activity, (ii) diel activity depends on acoustic communication, and (iii) both traits depend on each other. For each comparison, the AIC of the best-fitting model is boldfaced. The likelihood-ratio test compares the fit of the model of dependent evolution to the null model of independent evolution in both traits. Results shown here assumed different transition rates for gains and losses (ARD model) for both characters. Results based on the equal-rates model (ER) are similar and are shown in Supplementary Table 3

**Table 3:** Results of phylogenetic logistic regression testing the relationships between diel activity and the presence of acoustic communication.

Tree	Predictor	Alpha	Standard error	*P* value
Ericson	Maximum diurnal	0.2890	0.0838	0.0006
Hackett	Maximum diurnal	0.2890	0.0837	0.0006
Ericson	Maximum nocturnal	0.2567	0.1088	0.0184
Hackett	Maximum nocturnal	0.2567	0.1087	0.0182

Analyses were conducted using two different trees (Ericson^29^ vs. Hackett^30^ backbone trees for birds) and two methods for coding day–night activity patterns in arrhythmic and crepuscular species (maximum diurnal and maximum nocturnal). Alpha is the phylogenetic correlation parameter estimate. The standard error for each alpha estimate is also given.

### Acoustic communication and diversification

The presence of acoustic communication did not significantly influence diversification rates across tetrapod phylogeny. Specifically, among the five models tested, the HiSSE Null4 model had the strongest support based on the size-corrected AIC (AICc; Supplementary Table 5). Under this model, mean diversification rates (speciation − extinction; *r* = 0.08 events per million years (Myr^−1^)) were equal between lineages with and without acoustic communication for both trees (Supplementary Table 6). Models in which acoustic communication influenced diversification had much weaker support (with AICc differences > 35).

### Phylogenetic conservatism in acoustic communication

Our results showed strong phylogenetic signal in the presence of acoustic communication across tetrapods, with Pagel’s^26^ lambda close to the maximum of 1 (0.9643 and 0.9646 using the Ericson^29^ and Hackett^30^ avian backbone trees^31^, respectively). The best-fitting model for both trees was the estimated lambda model (Supplementary Table 7), with AIC differences >1900 relative to the model with no phylogenetic signal. The *D*-statistic^27^ also supported strong phylogenetic conservatism, with negative *D*-values (−0.4847 and −0.4971 for each tree). This test failed to reject a model with strong phylogenetic signal (>0.99) and significantly rejected a model without phylogenetic signal (*P* < 0.0001, *n* = 1799).

### Large-scale evolution of acoustic communication

**Fig. 1:**
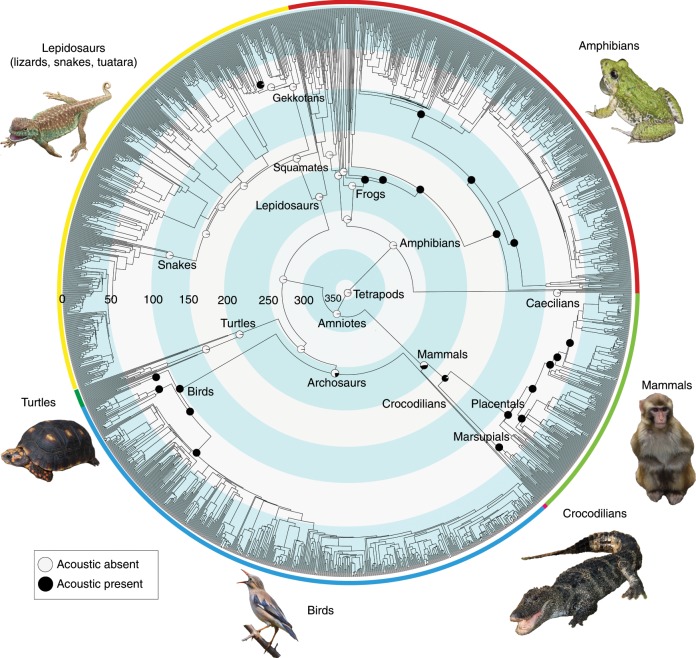
Summary of the evolution of acoustic communication across tetrapods. Pie diagrams at select nodes indicate proportional likelihoods of each state, with acoustic communication present (black) or absent (white). The tree includes 1799 species and is based on the Hackett^30^ backbone tree within birds. Reconstructions were based on the ARD model (with different rates for gains and losses). Major clades are indicated by colored rings on the outside of the tree; concentric circles (and associated numbers) indicate clade ages in millions of years before present^22^. Images of representative species are from Aijing Li, Jundong Tian, Xiaofei Zhai, Xiaowei Hong, and Yanjun Zhu. Reconstructions for all nodes and using alternative models and trees are shown as Supplementary Data 5–8 (this tree and model correspond to Supplementary Data 8). Source data are provided in Supplementary Data 3.

The evolution of acoustic communication across tetrapod phylogeny is summarized graphically in Fig. 1 (full results in Supplementary Data 5–8). The rate of origin of acoustic communication was higher than the rate of losses (0.00049 vs. 0.00028 Myr^−1^; both trees), given a model with different rates of gains and losses. However, a model with equal rates of gain and loss had similar fit (Supplementary Table 1).

Overall, we found that acoustic communication most likely evolved independently in each major tetrapod group, but its origins were ancient within many major clades (~100–200 Myr ago). Acoustic communication evolved early in the phylogeny of anurans but is absent in the sister group to all other living frogs (the clade containing Ascaphidae and Leiopelmatidae). The reconstruction for the root of all mammals is ambiguous (given variation among monotremes), but acoustic communication is strongly supported as present in the ancestor of therian mammals (marsupials + placentals) and placentals. In both frogs and mammals, acoustic communication evolved ~200 Myr ago and appears to have been maintained in many lineages to the present day. However, acoustic communication also appears to have been secondarily lost in several mammals and frogs (but not in birds).

Acoustic communication was inferred to be present in the most recent ancestor of living birds and the most recent ancestor of living crocodilians. Each of these ancestors is ~100 Myr old. It is possible that it was also present in the common ancestor of these two clades (~250 Myr old), but the reconstruction for this node is ambiguous.

Acoustic communication is rare among lepidosaurs but appears to have evolved repeatedly within the predominantly nocturnal clade, Gekkota. Acoustic communication evolved more recently in a few turtles and salamanders, among phylogenetically isolated species.

## Discussion

Acoustic communication is an intriguing and intensely studied aspect of animal behavior. However, research on the evolution of acoustic communication generally focuses on variation in acoustic signals within species and among close relatives. Here we address basic questions about the origins of acoustic communication across tetrapods, including relatively deep timescales (up to ~350 Myr ago). We find that the origins of acoustic communication are significantly associated with nocturnal activity. We also find that acoustic communication and diversification rates appear to be uncoupled across tetrapod lineages. We find that the presence of acoustic communication is strongly conserved phylogenetically. Finally, we find that acoustic communication evolved independently in most tetrapod groups but had relatively deep origins (~100–200 Myr ago) in major clades, including birds, crocodilians, frogs, and mammals. We discuss the significance of these findings, along with potential caveats, in the paragraphs below.

Our results show that the origins of acoustic communication are significantly associated with nocturnal activity. This hypothesis has previously been suggested^10^ but not tested. Our results make intuitive sense, given the independent origins of acoustic communication in lineages that have been reconstructed as ancestrally nocturnal, including frogs, salamanders, mammals, gekkotan lizards, and crocodilians^22^. The presence of acoustic communication in birds, which are predominantly diurnal, is more perplexing. However, many birds sing predominantly at dawn^32,33^, rather than in daylight. Furthermore, acoustic communication may have evolved in the common ancestor of crocodilians and birds (Fig. 1), which is reconstructed as nocturnal^22^, or before the origins of diurnal activity in modern birds. Similarly, acoustic communication is present in many other diurnal lineages that almost certainly evolved from nocturnal ancestors (e.g., dendrobatid frogs, primates^22^). Our results support the idea that acoustic signals evolved to allow communication at night, when most visual signals are less effective^10^. Yet, acoustic communication may remain useful in diurnal species, and so there may not be strong selective pressure to lose this trait in diurnal lineages once it has evolved. We do find cases where acoustic communication appears to have been lost evolutionarily, especially in frogs and mammals (Supplementary Data 5–8). Intriguingly, among species sampled in our study, acoustic communication appears to have been lost in both nocturnal frogs (*Acanthixalus*) and diurnal frogs (*Megaelosia*) but only in nocturnal mammal lineages (including the marsupials *Cercartetus* and *Tarsipes*, pangolins, and the rodents *Abrocoma* and *Jaculus* and various heteromyid genera). Yet, acoustic communication has been retained in the many mammal lineages that evolved diurnality (e.g., meerkats, pika, hyrax, and various artiodactyls, primates, and rodents). Similarly, we found no cases where acoustic communication was lost in birds.

The relationship between diel activity and acoustic communication found here supports the idea that signal evolution is strongly influenced by ecology. More specifically, the signaling environment where a species occurs is thought to strongly influence the signals that they evolve^11,12^. However, this idea is generally applied to different variants of a signal among closely related species (e.g., color among Rift-lake cichlids^13^). Our results suggest that this idea might help explain the origins of different types of signals (e.g., acoustic vs. visual) over timescales of hundreds of millions of years.

We find no association between rates of diversification and the presence of acoustic communication. This result is surprising, given evidence that divergence in acoustic signals among populations may play a role in speciation, at least in birds^5^ and frogs^3,4^. However, to our knowledge, previous studies have not actually shown that the presence of acoustic communication increases speciation rates. Indeed, given that acoustic communication is so widespread in these groups, the relationship between diversification and acoustic communication calls for testing at a broader phylogenetic scale (i.e., not within frogs or birds). Thus the scale should allow comparison among major vertebrate clades. Our analyses at this scale do not support the idea that acoustic communication drives diversification. Although this result is unexpected, consideration of the net diversification rates of these major clades helps explain why it occurs. For example, although birds have a high net diversification rate relative to other tetrapod clades, their living sister group (crocodilians) also has acoustic communication but has a low net diversification rate^34^. Similarly, lepidosaurs mostly lack acoustic communication (Fig. 1) but have a relatively high diversification rate overall^34^. Intriguingly, nocturnal activity seems to be associated with lower diversification rates than diurnal activity in tetrapods^22^. This effect, and the association between acoustic communication and nocturnal activity, might tend to counteract positive impacts of acoustic communication on diversification. Clearly, there is considerable variation in diversification rates among and within these clades that is not directly related to acoustic communication^34^. Furthermore, we coded the presence of acoustic communication without regard to its function within each species. Therefore, diversification rates might be higher in lineages in which acoustic signals are used specifically in mate choice and/or species recognition (e.g., birds, frogs). In contrast, acoustic communication in groups with lower diversification rates (e.g., crocodilians, mammals) may not be involved in mate choice and species recognition and so may be less relevant to speciation. However, there is evidence that acoustic signals can potentially be important for speciation in (at least some) mammals^35^. It is also possible that acoustic communication is important for maintaining distinct lineages without hybridization but is not necessarily the main driver of the formation of new lineages. Earlier studies have shown that strong links between sexually selected traits, speciation, and large-scale diversity patterns are only sometimes supported^19–21^. Our results support the idea that these linkages can be weak or absent and show this pattern at a much larger phylogenetic scale than most previous studies.

We also reconstructed the evolution of acoustic communication across tetrapod phylogeny (Fig. 1). There have been few (if any) previous analyses at this scale, despite an excellent review lacking explicit analyses^36^. Our reconstructions show that acoustic communication has arisen repeatedly and independently across major tetrapod groups (e.g., frogs, mammals, geckos, birds, crocodilians) and had remarkably ancient origins within these groups (~100–200 Myr ago). Our results also show that the presence of acoustic communication can be quite stable over these deep timescales, with strong conservatism and relatively few losses (at least among the sampled species). For example, we found only a handful of losses in frogs and mammals and none in birds. We speculate that some other types of signaling traits might be far more unstable and evolutionarily short-lived (e.g., conspicuous coloration, enlarged structures). Our results here provide a baseline for making quantitative comparisons and tests across different types of signaling traits.

Finally, we recognize that readers might have several concerns about the methods used here. First, how can we say acoustic communication is actually absent in a given species and not simply unreported? Although this is certainly a possibility in some species, it seems unlikely to have strongly influenced our overall results. For example, we recorded acoustic communication as generally present across mammals, birds, frogs, and crocodilians. If it were present in additional species in these groups, our main conclusions should be unchanged. The groups in which acoustic communication was found to be largely absent (turtles, salamanders, non-gekkotan lepidosaurs) are broadly considered to lack this trait, and its absence in most species should be uncontroversial. Second, can we accurately reconstruct ancestral states at such deep timescales? In fact, the absolute timescale is not necessarily critical to the accuracy of likelihood reconstructions. Instead, phylogenetic patterns of trait variability should be more relevant, and our results show that acoustic communication is strongly conserved phylogenetically. Previous studies have unambiguously reconstructed traits at nodes >500 Myr old^37^, yielding reconstructions consistent with fossil evidence. Most importantly, our reconstructions here are broadly consistent with a review that incorporated paleontological data^36^. Third, can we infer diversification rates and trait evolution with so few species sampled in the trees? HiSSE (and related approaches) for inferring diversification can be accurate with incomplete sampling^38^, and the absolute number of tips here is large. Indeed, HiSSE analyses with nearly identical trees show that these trees are large enough to strongly support trait-associated diversification (for diel activity^22^). Furthermore, our other phylogenetic tests yielded significant results, showing that incomplete taxon sampling did not prevent us from finding significant patterns of trait correlation and conservatism (Tables 2 and 3). It is very unclear how incomplete sampling alone would cause these tests of correlation and conservatism to yield incorrect but statistically significant results, especially given our relatively comprehensive sampling of clades and our proportional sampling of species within clades. Finally, an effect of acoustic communication on diversification might go undetected here if it were weak or variable among clades. Our test addresses whether there is a consistently strong effect across tetrapod clades. Overall, we acknowledge that there are many potential sources of error in our study, but none seem likely to overturn our main conclusions.

In summary, our study has revealed the large-scale patterns of evolution in acoustic communication in tetrapods. We show that origins of acoustic communication are associated with nocturnal activity patterns and that acoustic communication does not increase diversification rates. We find that acoustic communication evolved repeatedly across tetrapods, but had ancient origins in many groups, and is strongly conserved. Our results raise many questions for future research. For example, why did some nocturnal lineages evolve acoustic communication but not others (e.g., most salamanders, snakes)? Are there specific types of acoustic traits that increase diversification rates in particular clades^19^, if not acoustic communication in general? Are these patterns in tetrapods repeated in other clades with acoustic communication (e.g., insects, fish)?

## Methods

### Phylogeny

Our sampling of species for acoustic communication was guided by which species were included in time-calibrated trees and had diel activity data. Time calibration is essential for combining trees, estimating diversification rates, and reconstructing trait evolution. We started with a tetrapod phylogeny that included 1824 species^22^. This tree had four main advantages. First, all species already had diel activity data. Second, all major tetrapod clades were included (i.e., amphibians, mammals, birds, lepidosaurs, turtles, and crocodilians), with species sampled within each clade in proportion to the clade’s overall species richness (see details below). Proportional sampling is important for analyses relating traits and diversification. The sampling of species within these major clades was also designed to represent major groups (e.g., orders, families), with sampling roughly proportional to their species richness. The tree was generated by combining trees from studies focused on each major clade (amphibians^39^, birds^31^, crocodilians^40^, lepidosaurs^41^, mammals^42^, turtles^43^), linked by an overall phylogeny among major clades^44^. This latter tree has an estimated topology and divergence times that are similar to those from an extensive phylogenomic study^45^ but has better sampling of major clades. There were two versions of the overall tree^22^, differing in their backbone trees within birds (i.e., Ericson^29^ and Hackett^30^ trees^31^). Both trees were used for all analyses here. Third, although the tree has extensive taxon sampling, it is not so large as to make computationally intensive analyses impractical (e.g., HiSSE). Fourth, because the phylogeny was assembled for analyzing diel activity, the species sampling should be unbiased regarding whether species have or lack acoustic communication. Note that species sampling in the diel activity dataset was not designed to favor any particular diel activity state but rather to represent higher taxa (e.g., classes, orders, families) in proportion to their species richness (see above). The trees are available as Supplementary Data 1 and 2.

We recognize that there are more recent phylogenies available within some groups. However, many of these have more limited taxon sampling. Furthermore, we found the presence of acoustic communication to be largely invariant within most major clades (e.g., birds; Table 1). Therefore, alternative phylogenies within these groups should have limited impact on the overall results.

### Acoustic communication data

We obtained acoustic communication data from books, original papers, and online databases (e.g., xeno-canto: https://www.xeno-canto.org/). Data on individual species and supporting references are given in Supplementary Data 3. We searched the literature for data on each species in the tree using Web of Science and Google Scholar between December 1, 2017 and January 15, 2019. The following keywords were used: “acoustic communication,” “call,” “vocal communication,” “vocalization,” “song,” and “sound.” Each keyword was used in combination with the species name for every search.

Acoustic communication can be defined as the transmission of intraspecific messages via airborne sound waves^46^. We assigned species to one of the two states following this definition (0 for absence of acoustic communication, 1 for presence). Species that produce sound but are not known to exchange information from sound with conspecifics were considered to lack acoustic communication (e.g., rattling in rattlesnakes). Vibrations transmitted through a fluid (air or water) are generally defined as “sound,” whereas those in solids are generally referred to as “vibrations”^47^. Species utilizing only substrate-borne vibration communication were also characterized as lacking acoustic communication.

We recognize that there may be some cases in which sounds are produced but communication with conspecific individuals is present but not yet documented. We also recognize that conclusively documenting the absence of a behavior is challenging. We address why our conclusions should not be an artifact of these factors in the “Discussion” section.

There were 25 species initially included in the trees for which we could not find adequate data on the presence or absence of acoustic communication. These were pruned from the trees using Mesquite^48^ version 3.51. We avoided removing two mammal species by replacing species lacking data on acoustic communication data with congeners having these data. Specifically, we replaced *Heteromys anomalus* with *H. desmarestianus* and *Liomys adspersus* with *L. pictus*.

We obtained data for a total of 1799 tetrapod species (Table 1). We assembled estimates of the total described species richness of amphibians^49^, non-avian reptiles^50^, birds^51^, and mammals^52^. Based on these estimates, our sampling within clades was strongly proportional to each clade’s total richness (*r*^2^ = 0.936; *P* = 0.0016). Nevertheless, amphibians were somewhat oversampled relative to other clades (Table 1). However, described amphibian richness is still increasing rapidly, with ~700 species added in the past 3 years^49^ (2016–2019). Thus the actual number of amphibian species might be similar to the numbers of described lepidosaurs and birds.

### Testing relationships between acoustic communication and diel activity

We used diel activity data assembled in a previous study^22^, following their definitions. In brief, species were classified as being diurnal (primarily active by day), nocturnal (primarily active by night), crepuscular (active primarily at twilight and/or early morning), or arrhythmic (cathemeral; similarly active during the day and night or changing day–night activity seasonally). Many analyses required treating diel activity as a two-state character, and most tetrapod species^22^ seem to be primarily nocturnal (41%) or diurnal (51%). Therefore, they alternatively coded all crepuscular and arrhythmic species as either nocturnal or diurnal. Under “maximum diurnal” coding, crepuscular and arrhythmic species were coded as diurnal. Under “maximum nocturnal” coding, these species were coded as nocturnal. We used these strategies here, given that our analyses also require two-state coding. Diel activity data are given in Supplementary Data 4.

We used two approaches to test whether the presence of acoustic communication is associated with nocturnal activity. First, we compared maximum-likelihood models of independent and dependent evolution between diel activity and acoustic communication^23^. These models test how one trait affects the transition rates of the other trait. Analyses were performed using the R package *phytools*^53^ version 0.6-60. We primarily tested whether acoustic communication depends on diel activity, but we also tested whether diel activity depends on acoustic communication, and whether both depend on each other. Prior to these analyses, we compared the fit of models with equal transition rates (i.e., gains and losses, 0 to 1 and 1 to 0, respectively) between states of each character (ER) and models with all transition rates different between states (ARD). Models were compared with the function fitDiscrete in *GEIGER*^54^ version 2.0 using AIC values (lowest AIC considered best-fitting^28^). For maximum diurnal coding, the best fit model was ARD, but AIC differences between models for maximum nocturnal coding were <4 (Supplementary Table 2). For acoustic communication, AIC differences were also <4 (Supplementary Table 1). Therefore, we used both ARD and ER models for both variables for these tests. Likelihood-ratio tests were used to evaluate whether a model of correlated evolution explained the data better than the null model of independent evolution of the two traits. In addition, models were compared using the AIC and AIC_w_ statistics^28^. The AIC_w_ measures the weight of evidence in favor of a model, on a scale from 0 to 1. The model with the highest AIC_w_ had the best fit.

For the second approach, we used phylogenetic logistic regression^24^. This approach can be used to test for relationships between two categorical variables. We utilized the function phyloglm in the R package *phylolm*^55^ version 2.6. We used the IG10 phylogenetic generalized linear model^24^. This method modifies a GLM (generalized linear model) framework for binomial distributions to incorporate a correlation matrix representing relationships among taxa. We treated diel activity as the independent variable and acoustic communication as the dependent variable.

### Diversification

To test whether acoustic communication increased diversification rates, we used the BiSSE (Binary State Speciation and Extinction) and HiSSE approaches in the R packages *diversitree*^56^ version 0.9–7 and *HiSSE*^57^ version 1.8. Importantly, HiSSE incorporates unmeasured factors (i.e., hidden states) that could impact diversification rates besides the included character. We compared the fit of five different models. We first ran two BiSSE models: (i) a full BiSSE model with two observed states (acoustic communication absent or present, 0 and 1, respectively), with different rates of transitions (*q*_01_, *q*_10_), speciation (*λ*_0_, *λ*_1_), and extinction (*μ*_0_, *μ*_1_) associated with each state. (ii) Second, a restricted BiSSE-equivalent model^57^ with two observed states and no hidden states, constrained speciation and extinction rates (*λ*_0_ = *λ*_1_, *μ*_0_ = *μ*_1_), and two transition rates (HiSSE two-state model). We then ran a full HiSSE model (iii) with two hidden states (A, B) contained within each observed state (i.e., states 0A, 1A, 0B, 1B), speciation and extinction rates varying independently across all four states, and transition rates between all observed and hidden states free to vary except for dual transitions (e.g., q1A to q0B, q0A to q1B). These dual transitions were considered unlikely and were disallowed^57^. In addition, we tested two null HiSSE models: (iv) HiSSE Null2 (two hidden states), with different speciation and extinction rates only between the hidden states and different transition rates between observed and hidden states, and (v) HiSSE Null4 (four hidden states) with two additional hidden states (C, D), different speciation and extinction rates for each hidden state but not for the observed states, and equal transition rates across all the hidden and observed states. The first model was implemented in *diversitree*, and models ii–v were implemented in *HiSSE*. All analyses were conducted with default root setting “madfitz”^38^. Comparisons were conducted for both trees. Models were evaluated based on AICc scores^28^.

Note that BiSSE/HiSSE analyses can account for incomplete sampling of species by correcting for biased sampling of states among species^38^. However, we considered our sampling of states among species to be unbiased, and so no correction was considered necessary.

### Testing for phylogenetic signal

Prior to reconstructing the evolution of acoustic communication across the tree, we evaluated whether this trait was phylogenetically conserved. Phylogenetic signal was first evaluated using Pagel’s^26^ lambda test, utilizing the fitDiscrete function in *GEIGER*^54^. Low lambda values (close to 0) indicate weak phylogenetic signal, whereas higher values (close to 1) indicate strong signal^26^. In addition, we also compared the fit of a model based on the estimated lambda value (EL) to a white-noise (WN) model, with no phylogenetic signal. Likelihoods were compared using the AIC. AIC differences >4 between models were considered strong support for the best-fitting model^28^.

Phylogenetic signal was also assessed using the *D*-statistic^27^. The *D*-statistic was designed to test for phylogenetic signal in binary traits. We used the phylo.d function in the R package *caper*^58^ version 0.5.2. The *D*-statistic compares the observed *D*-value to alternative *D*-values generated with simulated data based on Brownian motion (BM; strong phylogenetic signal) and WN models (no signal). Negative *D*-values indicate traits are strongly clumped (i.e., more conserved than BM), *D* = 0 supports the BM model, and *D* = 1 supports the WN model^27^. We also tested whether the *D*-statistic differed significantly from the BM and WN models. Non-significant *P* values for BM indicate conserved trait evolution. Note that phylogenetic signal will reflect low evolutionary rates (and phylogenetic conservatism) when analyzing discrete variables^59^, as done here.

### Ancestral-state reconstruction

We reconstructed the evolution of acoustic communication across the tree using maximum-likelihood ancestral-state reconstruction. Different diversification rates associated with different states can potentially impact reconstructions^60^. However, the HiSSE Null4 model had the strongest support for both trees, suggesting that acoustic communication did not significantly impact diversification rates (Table S5). Therefore, we used simpler models that did not incorporate diversification (ARD and ER, see above). Ancestral reconstructions were performed using both the ARD and ER models, given the similar fit between these models (Table S1).

### Reporting summary

Further information on research design is available in the Nature Research Reporting Summary linked to this article.

## Supplementary information

Supplementary Information

Reporting Summary

Description of Additional Supplementary Files

Supplementary Data 1

Supplementary Data 2

Supplementary Data 3

Supplementary Data 4

Supplementary Data 5

Supplementary Data 6

Supplementary Data 7

Supplementary Data 8

## Data Availability

Data are available as Supplementary Data 1–8 and on Dryad (10.5061/dryad.r4xgxd288)^61^.

## Code availability

This study used available R packages rather than custom coding.
