# Supplementary material for: The origins of acoustic communication in vertebrates
Source: Nat Commun. 2020 Jan 17;11:369. doi: 10.1038/s41467-020-14356-3 (PMC6969000; doi:10.1038/s41467-020-14356-3)
Supplement: Supplementary file 1 — Supplementary Information [file 41467_2020_14356_MOESM1_ESM.pdf]

Supplementary Information

The origins of acoustic communication in vertebrates

Chen and Wiens

Table of Contents

Supplementary Table 1. Comparison of the fit of different models for the evolution of acoustic communication.

Supplementary Table 2. Comparison of the fit of different models for the evolution of diel activity.

Supplementary Table 3. Results of likelihood analyses of correlated evolution between acoustic communication and diel activity.

Supplementary Table 4. Results of likelihood analyses of correlated evolution between acoustic communication and diel activity, comparing AIC and AICw from different models.

Supplementary Table 5. Comparison of the fit of different HiSSE models relating acoustic communication and diversification.

Supplementary Table 6. Estimated diversification parameters of the best-fitting HiSSE model.

Supplementary Table 7. Testing for phylogenetic signal in the evolution of acoustic communication.

Supplementary References

Supplementary Table 1. Comparison of the fit of different models for the evolution of acoustic communication. Results are shown for different trees based on different higher-level backbone phylogenies within birds (Ericson[1] vs. Hackett[2]). Models are ARD (all rates different, with different rates for 0 to 1 vs. 1 to 0 transitions) and ER (equal rates, single transition rate for 0 to 1 and 1 to 0 transitions). ML=maximum likelihood of model. AIC=Akaike information criterion. ΔAIC=difference between AIC of a given model and the best fitting model for the data (boldfaced) for each tree.

	Model	ML	AIC	ΔAIC
Ericson	ARD	-138.9986	281.9972	0.2048
	ER	-139.8962	**281.7924**	0.0000
Hackett	ARD	-138.7976	281.5951	0.2883
	ER	-139.6534	**281.3068**	0.0000

Supplementary Table 2. Comparison of the fit of different models for the evolution of diel activity. Two different approaches were used to code species arrhythmic and crepuscular species, either treating them as diurnal (maximum diurnal coding) or nocturnal (maximum nocturnal coding). Results are shown for different trees based on different higher-level backbone phylogenies within birds (Ericson[1] vs. Hackett[2]). Models are ARD (all rates different, with different rates for 0 to 1 vs. 1 to 0 transitions) and ER (equal rates, single transition rate for 0 to 1 and 1 to 0 transitions). ML=maximum likelihood of model. AIC=Akaike information criterion. ΔAIC=difference between the AIC of a given model and the best model for the data (boldfaced) for the tree.

Coding and tree	Model	ML	AIC	ΔAIC
Maximum diurnal				
Ericson	ARD	-690.4638	**1384.9276**	0.0000
	ER	-699.7949	1401.5898	16.6622
Hackett	ARD	-689.1796	**1382.3592**	0.0000
	ER	-698.6795	1399.3590	16.9998
Maximum nocturnal				
Ericson	ARD	-554.9681	1113.9361	1.5748
	ER	-555.1795	**1112.3613**	0.0000
Hackett	ARD	-554.9722	1113.9445	1.6792
	ER	-555.1315	**1112.2653**	0.0000

Supplementary Table 3. Results of likelihood analyses of correlated evolution between acoustic communication and diel activity. Analyses used the equal rates (ER) model for both traits, with a single rate for gains and losses. Results using the all-rates different model (ARD) for both traits are similar (Table 2). Analyses used two methods for coding diel activity in arrhythmic and crepuscular species (maximum diurnal, maximum nocturnal) and two trees (Ericson[1], Hackett[2]). The AIC of the best-fitting model is boldfaced. The likelihood-ratio test compares the fit of the model of dependent evolution to the null model of independent evolution in both traits. Different analyses assumed different traits were dependent.

Coding and tree	Dependent variable	Model	Independent model		Dependent model		Likelihood ratio (LRT)	*P*
			Log-likelihood	AIC	Log-likelihood	AIC		
Maximum diurnal								
Ericson	Acoustic	ER	-839.7687	1683.537	-833.8880	**1673.776**	11.7615	0.0006
	Diel	ER	-839.7687	**1683.537**	-839.2126	1684.425	1.1123	0.2916
	Acoustic & diel	ER	-839.7687	1683.537	-833.3335	**1674.667**	12.8704	0.0016
Hackett	Acoustic	ER	-838.4126	1680.825	-832.6367	**1671.273**	11.5516	0.0007
	Diel	ER	-838.4126	**1680.825**	-837.8241	1681.648	1.1768	0.2780
	Acoustic & diel	ER	-838.4126	1680.825	-832.0472	**1672.094**	12.7307	0.0017
Maximum nocturnal								
Ericson	Acoustic	ER	-695.5589	1395.118	-688.8989	**1383.798**	13.3200	0.0003
	Diel	ER	-695.5589	**1395.118**	-695.5582	1397.116	0.0013	0.9709
	Acoustic & diel	ER	-695.5589	1395.118	-688.8666	**1385.733**	13.3847	0.0012
Hackett	Acoustic	ER	-695.2746	1394.549	-688.7746	**1383.549**	12.9999	0.0003

	Diel	ER	-695.2746	**1394.549**	-695.2740	1396.548	0.0011	0.9735
	Acoustic & diel	ER	-695.2746	1394.549	-688.7425	**1385.485**	13.0642	0.0015

Supplementary Table 4. Results of likelihood analyses of correlated evolution between acoustic communication and diel activity, comparing AIC and AIC_W from different models. These analyses address whether the evolution of these traits is independent of each other (independent column), whether the origin of acoustic communication depends on diel activity (acoustic dependent column), whether diel activity depends on acoustic communication (diel dependent), or whether both traits dependent on each other (both dependent). Results are based on different coding methods for diel activity (maximum diurnal, maximum nocturnal), different trees (Ericson[1], Hackett[2]), and different models for rates of change between states (ARD: all-rates different; ER: equal rates).

Coding and tree	Model	AIC/ AIC_W	Independent	Diel dependent	Acoustic dependent	Both dependent
Maximum diurnal						
Ericson	ARD	AIC	1667.669	1671.599	**1656.630**	1660.581
	ARD	AIC_W	0.0035	0.0005	**0.8747**	0.1213
	ER	AIC	1683.537	1684.425	**1673.776**	1674.667
	ER	AIC_W	0.0046	0.0030	**0.6050**	0.3875
Hackett	ARD	AIC	1679.120	1682.932	**1668.694**	1672.662
	ARD	AIC_W	0.0048	0.0007	**0.8743**	0.1202
	ER	AIC	1680.825	1681.648	**1671.273**	1672.094
	ER	AIC_W	0.0050	0.0033	**0.5962**	0.3955
Maximum nocturnal						
Ericson	ARD	AIC	1396.779	1400.731	**1384.295**	1388.039
	ARD	AIC_W	0.0017	0.0002	**0.8650**	0.1331
	ER	AIC	1395.118	1397.116	**1383.798**	1385.733
	ER	AIC_W	0.0025	0.0009	**0.7222**	0.2744
Hackett	ARD	AIC	1410.804	1414.757	**1395.390**	1399.124
	ARD	AIC_W	0.0004	0.0001	**0.8657**	0.1338
	ER	AIC	1394.549	1396.548	**1383.549**	1385.485
	ER	AIC_W	0.0030	0.0011	**0.7218**	0.2742

Supplementary Table 5. Comparison of the fit of different HiSSE models relating acoustic communication and diversification. Analyses were conducted using two trees (with the Ericson[1] vs. Hackett[2] backbone trees for birds). The model with the lowest AICc (boldfaced) has the best fit. The ΔAICc is the difference between a given model and the best-fitting model. The full BiSSE model allows different parameters for each of the two observed states, with no hidden states. The two-state HiSSE model is equivalent to a constrained BiSSE model with no hidden states, with shared speciation and extinction rates between the two observed states (0 and 1, acoustic communication absent and present), and with two transition rates between the two observed states. The full HiSSE model has two observed and two hidden states, with different speciation and extinction rates possible for each of the four combinations of hidden and observed states (0A, 0B, 1A, 1B). The Null2 model pairs each observed state with two hidden states (A, B) but constrains speciation and extinction rates to be the same between the hidden states in each observed state (i.e. 1A=0A). Thus, only the hidden states can have different rates of speciation and extinction, but not the observed states. The Null4 model allows for eight pairs of two observed states (0, 1) and four hidden states (A, B, C, D) with speciation and extinction rates constrained across hidden states, such that only the four hidden states can each have different rates of speciation and extinction and the observed states do not. Only results from the same tree are comparable. Best fitting models are highlighted in bold. The strong support for the "HiSSE Null 4" model suggests that hidden states (not acoustic communication) have the strongest impact on diversification. Note that strong support for the "HiSSE Full" model instead would suggest that both hidden states and acoustic communication influenced diversification.

Tree	Model	ML	AICc	ΔAICc
Ericson				
	BiSSE Full	-8492.16	16996.31	781.83
	HiSSE Two-State	-8525.44	17058.91	844.43

	HiSSE Full	-8112.64	16265.76	51.28
	HiSSE Null2	-8542.47	17094.98	880.50
	**HiSSE Null4**	**-8098.19**	**16214.48**	**0.00**
Hackett				
	BiSSE Full	-8470.76	16953.52	801.88
	HiSSE Two-State	-8508.08	17024.17	872.53
	HiSSE Full	-8074.83	16190.14	38.50
	HiSSE Null2	-8526.29	17062.62	910.98
	**HiSSE Null4**	**-8066.77**	**16151.64**	**0.00**

Supplementary Table 6. Estimated diversification parameters of the best-fitting HiSSE model. Parameters include rates of speciation (λ) and extinction (μ) for the observed states (acoustic communication absent and present, states 0 and 1) as well as the hidden states (A, B, C, D). The best-fitting HiSSE model is HiSSE Null4 for both trees (Supplementary Table 5). Rates of speciation (λ) and extinction (μ) are given in events per million years. The estimated net diversification rates (*r*, speciation-extinction) are also given across the four hidden states. For the Ericson tree, the mean diversification rate across hidden states for both observed states is 0.0753. For the Hackett tree, the mean diversification rate is 0.0756.

Tree and parameters	0A	0B	0C	0D	1A	1B	1C	1D
Ericson								
λ	0.0561	0.1112	0.0723	0.7227	0.0561	0.1112	0.0723	0.7227
μ	0.0000	0.0005	0.0582	0.6023	0.0000	0.0005	0.0582	0.6023
r	0.0561	0.1107	0.0141	0.1204	0.0561	0.1107	0.0141	0.1204
Hackett								
λ	0.0210	0.1003	0.0574	0.5296	0.0210	0.1003	0.0574	0.5296
μ	0.0011	0.0000	0.0000	0.4046	0.0011	0.0000	0.0000	0.4046
r	0.0199	0.1003	0.0574	0.1250	0.0199	0.1003	0.0574	0.1250

Supplementary Table 7. Testing for phylogenetic signal in the evolution of acoustic communication. These comparisons address whether the best-fitting model includes phylogenetic signal (estimated lambda: EL) or lacks phylogenetic signal (white noise: WN). Analyses were performed on both trees (Ericson[1] and Hackett[2] backbone trees within birds). The best-fitting model for each tree (lowest AICc) is boldfaced. ΔAICc is the difference between each model and the best-fitting model for that tree.

Tree	Model	Model likelihood	AICc	ΔAICc
Ericson tree	EL	-138.3241	**282.6616**	0.0000
	WN	-1111.6000	2225.2030	1942.5414
Hackett tree	EL	-145.3146	**296.6425**	0.0000
	WN	-1109.9810	2221.9640	1925.3215

Supplementary References

1. P. G. P. Ericson, C. L. Anderson, T. Britton, A. Eizanowski, U. F. Johansson, M. Kallersjo, J. I. Ohlson, T. J. Parsons, D. Zuccon, G. Mayr. Diversification of Neoaves: integration of molecular sequence data and fossils. *Biol. Lett.* **2**, 543–547 (2006).

2. S. J. Hackett, R. T. Kimball, S. Reddy, R. Bowie, E. L. Braun, M. J. Braun, et al. 2008. A phylogenomic study of birds reveals their evolutionary history. *Science* **320**, 1763–1768 (2008).
