# Supplementary material for: The origins of acoustic communication in vertebrates
Source: Nat Commun. 2020 Jan 17;11:369. doi: 10.1038/s41467-020-14356-3 (PMC6969000; doi:10.1038/s41467-020-14356-3)
Supplement: Supplementary file 2 — Reporting Summary [file 41467_2020_14356_MOESM2_ESM.pdf]

nature research

Corresponding author(s): John J. Wiens

Last updated by author(s): Dec 23, 2019

Reporting Summary

Nature Research wishes to improve the reproducibility of the work that we publish. This form provides structure for consistency and transparency in reporting. For further information on Nature Research policies, see Authors & Referees and the Editorial Policy Checklist .

Statistics

For all statistical analyses, confirm that the following items are present in the figure legend, table legend, main text, or Methods section.

n/a	Confirmed	
☐	☒	The exact sample size (*n*) for each experimental group/condition, given as a discrete number and unit of measurement
☒	☐	A statement on whether measurements were taken from distinct samples or whether the same sample was measured repeatedly
☐	☒	The statistical test(s) used AND whether they are one- or two-sided<br>*Only common tests should be described solely by name; describe more complex techniques in the Methods section.*
☒	☐	A description of all covariates tested
☐	☒	A description of any assumptions or corrections, such as tests of normality and adjustment for multiple comparisons
☐	☒	A full description of the statistical parameters including central tendency (e.g. means) or other basic estimates (e.g. regression coefficient) AND variation (e.g. standard deviation) or associated estimates of uncertainty (e.g. confidence intervals)
☐	☒	For null hypothesis testing, the test statistic (e.g. *F*, *t*, *r*) with confidence intervals, effect sizes, degrees of freedom and *P* value noted<br>*Give P values as exact values whenever suitable.*
☒	☐	For Bayesian analysis, information on the choice of priors and Markov chain Monte Carlo settings
☒	☐	For hierarchical and complex designs, identification of the appropriate level for tests and full reporting of outcomes
☒	☐	Estimates of effect sizes (e.g. Cohen's *d*, Pearson's *r*), indicating how they were calculated

Our web collection on statistics for biologists contains articles on many of the points above.

Software and code

Policy information about availability of computer code

Data collection	No software was used to collect the data.
Data analysis	Data analysis was conducted using the following R packages: caper version 0.5.2, diversitree version 0.9-7, Geiger version 2.0, caper version 1.01, phylolm version 2.6, and phytools version 0.6-60

For manuscripts utilizing custom algorithms or software that are central to the research but not yet described in published literature, software must be made available to editors/reviewers. We strongly encourage code deposition in a community repository (e.g. GitHub). See the Nature Research guidelines for submitting code & software for further information.

Data

Policy information about availability of data

All manuscripts must include a data availability statement. This statement should provide the following information, where applicable:
- Accession codes, unique identifiers, or web links for publicly available datasets
- A list of figures that have associated raw data
- A description of any restrictions on data availability

Data are available in the Supplementary Information as Datasets S1–S4.

Field-specific reporting

Please select the one below that is the best fit for your research. If you are not sure, read the appropriate sections before making your selection.

☐ Life sciences ☐ Behavioural & social sciences ☒ Ecological, evolutionary & environmental sciences

Ecological, evolutionary & environmental sciences study design

All studies must disclose on these points even when the disclosure is negative.

Study description	Our study analyzes the evolution of acoustic communication in terrestrial vertebrates.
Research sample	Our study combined data on acoustic communication, diel activity, and phylogeny from 1799 vertebrate species.
Sampling strategy	We included tetrapod species which had available data on diel activity and that were included in a time-calibrated phylogeny. We obtained data on acoustic communication for these species.
Data collection	We generated a dataset on the presence and absence of acoustic communication from the literature, using a systematic search.
Timing and spatial scale	We searched the literature for data on acoustic communication from December 1, 2017 and January 15, 2019. Species were distributed across the globe.
Data exclusions	Species lacking data on acoustic communication were excluded from the analyses.
Reproducibility	Our study is not based on experiments. It is based on macroevolutionary analyses of phylogenetic, diel activity and acoustic communication data.
Randomization	There was no random allocation into groups.
Blinding	Blinding was not relevant to our study.

Did the study involve field work? ☐ Yes ☒ No

Reporting for specific materials, systems and methods

We require information from authors about some types of materials, experimental systems and methods used in many studies. Here, indicate whether each material, system or method listed is relevant to your study. If you are not sure if a list item applies to your research, read the appropriate section before selecting a response.

Materials & experimental systems

n/a	Involved in the study	
☒	☐	Antibodies
☒	☐	Eukaryotic cell lines
☒	☐	Palaeontology
☒	☐	Animals and other organisms
☒	☐	Human research participants
☒	☐	Clinical data

Methods

n/a	Involved in the study	
☒	☐	ChIP-seq
☒	☐	Flow cytometry
☒	☐	MRI-based neuroimaging
