# Supplementary material for: The origins of acoustic communication in vertebrates
Source: Nat Commun. 2020 Jan 17;11:369. doi: 10.1038/s41467-020-14356-3 (PMC6969000; doi:10.1038/s41467-020-14356-3)
Supplement: Supplementary file 3 — Description of Additional Supplementary Files [file 41467_2020_14356_MOESM3_ESM.pdf]

Description of Additional Supplementary Files

File Name: Supplementary Data 1
Description: Phylogenetic tree for 1799 tetrapod taxa, using the Erickson backbone tree for birds.

File Name: Supplementary Data 2
Description: Phylogenetic tree for 1799 tetrapod taxa, using the Hackett backbone tree for birds.

File Name: Supplementary Data 3
Description: Data on acoustic communication in tetrapods.

File Name: Supplementary Data 4
Description: Data on diel activity in tetrapods.

File Name: Supplementary Data 5
Description: Evolution of acoustic communication across tetrapods, based on ancestral-state reconstructions from the ER model and the backbone tree of Ericson et al. (2006) for birds. Pie diagrams indicate proportional likelihoods of each state, with acoustic communication being present (blue) or absent (red). Pie diagrams at tips indicate the observed state in each sampled species.

File Name: Supplementary Data 6
Description: Evolution of acoustic communication across tetrapods, based on ancestral-state reconstructions from the ARD model and the backbone tree of Ericson et al. (2006) for birds. Pie diagrams indicate proportional likelihoods of each state, with acoustic communication being present (blue) or absent (red). Pie diagrams at tips indicate the observed state in each sampled species.

File Name: Supplementary Data 7
Description: Evolution of acoustic communication across tetrapods, based on ancestral-state reconstructions from the ER model and the backbone tree of Hackett et al. (2008) for birds. Pie diagrams indicate proportional likelihoods of each state, with acoustic communication being present (blue) or absent (red). Pie diagrams at tips indicate the observed state in each sampled species.

File Name: Supplementary Data 8
Description: Evolution of acoustic communication across tetrapods, based on ancestral-state reconstructions from the ARD model and the backbone tree of Hackett et al. (2008) for birds. Pie diagrams indicate proportional likelihoods of each state, with acoustic communication being present (blue) or absent (red). Pie diagrams at tips indicate the observed state in each sampled species.
