# Supplementary material for: The origins of acoustic communication in vertebrates
Source: Nat Commun. 2020 Jan 17;11:369. doi: 10.1038/s41467-020-14356-3 (PMC6969000; doi:10.1038/s41467-020-14356-3)
Supplement: Supplementary file 11 — Supplementary Data 8 [file 41467_2020_14356_MOESM11_ESM.pdf]

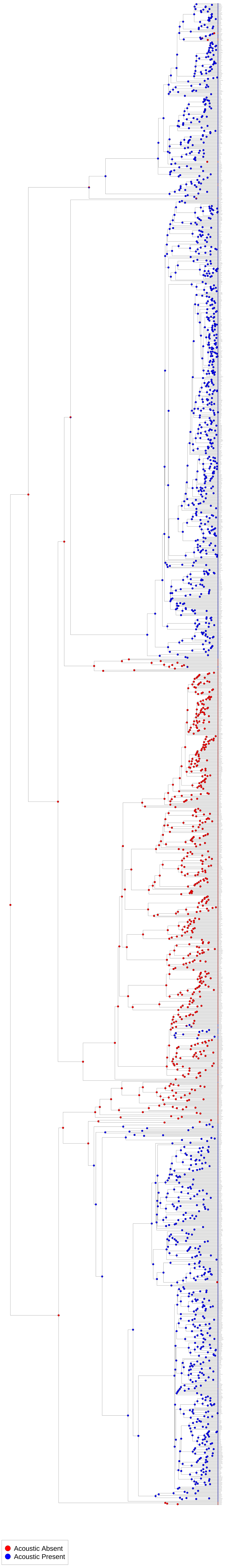
Acoustic Absent
Acoustic Present
